# Targeting Sirt1, AMPK, Nrf2, CK2, and Soluble Guanylate Cyclase with Nutraceuticals: A Practical Strategy for Preserving Bone Mass

**DOI:** 10.3390/ijms23094776

**Published:** 2022-04-26

**Authors:** Mark F. McCarty, Lidianys Lewis Lujan, Simon Iloki Assanga

**Affiliations:** 1 Catalytic Longevity Foundation, San Diego, CA 92109, USA; 2 Department of Research and Postgraduate in Food Science, Sonoran University, Hermosillo 83200, Mexico; lidianys1@yahoo.es; 3 Department of Biological Chemical Sciences, Sonoran University, Hermosillo 83200, Mexico; ilokiassanga@gmail.com

**Keywords:** osteoporosis, osteoblasts, osteoclasts, RUNX2, NFATc1, Sirt1, AMPK, Nrf2, soluble guanylate cyclase, nutraceuticals

## Abstract

There is a vast pre-clinical literature suggesting that certain nutraceuticals have the potential to aid the preservation of bone mass in the context of estrogen withdrawal, glucocorticoid treatment, chronic inflammation, or aging. In an effort to bring some logical clarity to these findings, the signaling pathways regulating osteoblast, osteocyte, and osteoclast induction, activity, and survival are briefly reviewed in the present study. The focus is placed on the following factors: the mechanisms that induce and activate the RUNX2 transcription factor, a key driver of osteoblast differentiation and function; the promotion of autophagy and prevention of apoptosis in osteoblasts/osteoclasts; and the induction and activation of NFATc1, which promotes the expression of many proteins required for osteoclast-mediated osteolysis. This analysis suggests that the activation of sirtuin 1 (Sirt1), AMP-activated protein kinase (AMPK), the Nrf2 transcription factor, and soluble guanylate cyclase (sGC) can be expected to aid the maintenance of bone mass, whereas the inhibition of the serine kinase CK2 should also be protective in this regard. Fortuitously, nutraceuticals are available to address each of these targets. Sirt1 activation can be promoted with ferulic acid, N1-methylnicotinamide, melatonin, nicotinamide riboside, glucosamine, and thymoquinone. Berberine, such as the drug metformin, is a clinically useful activator of AMPK. Many agents, including lipoic acid, melatonin, thymoquinone, astaxanthin, and crucifera-derived sulforaphane, can promote Nrf2 activity. Pharmacological doses of biotin can directly stimulate sGC. Additionally, certain flavonols, notably quercetin, can inhibit CK2 in high nanomolar concentrations that may be clinically relevant. Many, though not all, of these agents have shown favorable effects on bone density and structure in rodent models of bone loss. Complex nutraceutical regimens providing a selection of these nutraceuticals in clinically meaningful doses may have an important potential for preserving bone health. Concurrent supplementation with taurine, N-acetylcysteine, vitamins D and K2, and minerals, including magnesium, zinc, and manganese, plus a diet naturally high in potassium, may also be helpful in this regard.

## 1. Determinates of Bone Loss Post-Menopausally and with Aging and Inflammation

The loss of bone mass associated with an increased fracture risk is observed post-menopausally, with prolonged glucocorticoid therapy, during chronic inflammatory disorders, and with advancing age (senile osteoporosis). Post-menopausal bone loss primarily reflects an increase in osteolytic osteoclast activity, reflecting a loss of ERα-mediated estrogen activity that functions to suppress the production of the receptor activator of the NF-kB ligand (RANKL) by bone-lining cells [1]. RANKL is an agonist for the receptor activator of NF-kB (RANK) expressed by osteoclasts; the pre-exposure of osteoclast precursors to the macrophage colony-stimulating factor (M-CSF) is required for the expression of RANK [2]. The stimulation of RANK via RANKL is a key mediator of osteoclast maturation and activation [3]. Hence, the loss of estrogen activity up-regulates bone osteolytic activity owing to RANKL overproduction. In contrast, senile osteoporosis reflects a loss of the bone-forming capacity, owing to the decreased differentiation of mesenchymal stem cells into osteoblasts, coupled with the decreased survival or senescence of osteoblasts and osteocytes [4]. Although a direct contribution of osteocytes to bone formation is unclear, they play a crucial role in regulating the competing functions of osteoblasts and osteoclasts, and mediate the positive impact of mechanical loading on bone density; their excessive loss by apoptosis during estrogen withdrawal, glucocorticoid treatment, or aging is a key factor in the development of osteoporosis [5,6,7]. Osteocyte senescence is also a factor of bone loss with advancing age [8].

To ward off the loss of bone mass, a logical strategy is to promote the differentiation, function, and survival of osteoblasts and osteocytes, while concurrently suppressing the osteolytic activity of osteoclasts; the latter will be of particular importance in the context of the onset of menopause. With respect to osteoblasts, the RUNX2 transcription factor is the master regulator of osteoblast formation and function, driving the transcription of a number of genes essential for the bone forming process [9]. Hence, up-regulating the signaling pathways driving RUNX2 expression and activation can be expected to promote increased bone formation. The loss of bone-forming capacity associated with senile osteoporosis—and also, in some measure, estrogen deficiency—is characterized by increased apoptosis in osteoblasts and osteocytes; measures which suppress this apoptosis should also be useful. Additionally, osteoblast autophagy plays a key role in bone mineral deposition—autophagic vacuoles in osteoblasts secrete apatite crystals—while helping to ward off apoptosis and senescence in osteoblasts and osteocytes; hence, the up-regulation of autophagy in these cells is another key goal [10,11,12].

With respect to osteoclasts, the transcription factor nuclear factor of activated T cells c1 (NFATc1) is the primary driver of osteoclast maturation and activity; the down-regulation of NFATc1 expression and activation is therefore a key goal in osteoporosis prevention [3,13,14].

**Figure 1:**
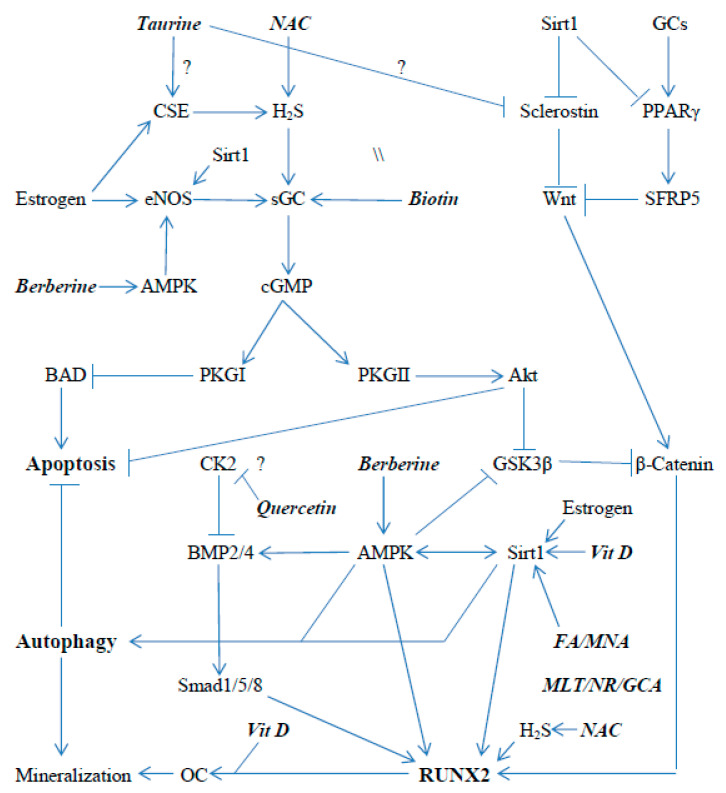
Nutraceutical mechanisms for the support of RUNX2 activity, promotion of autophagy, and inhibition of apoptosis in osteoblasts/osteocytes. NAC = N-acetylcysteine; FA = ferulic acid; MNA = N1-methylnicotinamide; MLT = melatonin; NR = nicotinamide riboside; and GCA = glucosamine.

**Figure 2:**
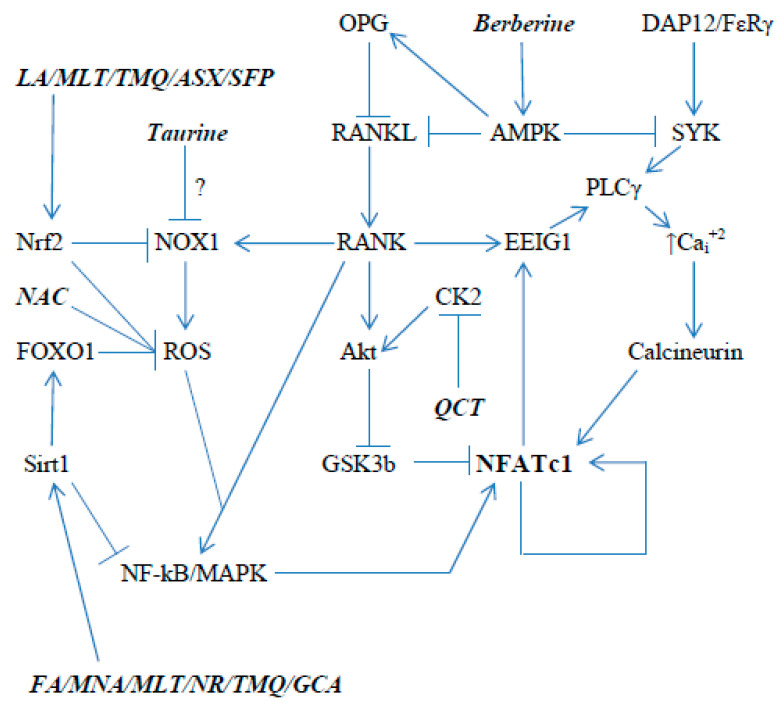
Nutraceutical modulation of osteoclast expression and activity of NFATc1, a key driver of osteolysis. LA = lipoic acid; MLT = melatonin; TMQ = thymoquinone; ASX = astaxanthin; SFP = sulforaphane; NAC = N-acetylcysteine; QCT = quercetin; FA = ferulic acid; MNA = N1-methylnicotinamide; NR = nicotinamide riboside; and GCA = glucosamine.

Figure 1 and Figure 2 schematically depict some of the signaling pathways in osteoblasts and osteoclasts that promote the expression and activity of RUNX2 and NFATc1, respectively; Figure 1 also displays pathways that regulate apoptosis and autophagy in osteoblasts/osteocytes. An analysis of these pathways, and of the research literature on osteoporosis, suggests that activation of AMP-activated protein kinase (AMPK), sirtuin 1 (sirt1), soluble guanylate cyclase (sGC), and the Nrf2 transcription factor, and the inhibition of the kinase CK2 could be expected to enhance the expression and activation of RUNX2 in osteoblasts, while promoting autophagy and inhibiting apoptosis in osteoblasts/osteocytes. Importantly, nutraceuticals with the potential to achieve each of these aims are available—as depicted in Figure 1. Analogously, the activation of AMPK, Sirt1, and Nrf2, and the inhibition of CK2, may be useful for decreasing the expression and activity of NFATc1 in osteoclasts.

## 2. A Brief Review of the Molecular Biology Determining Osteoblast and Osteoclast Activity

### 2.1. Regulation of RUNX2 Activity, Apoptosis, and Autophagy in Osteoblasts and Osteocytes

The major signaling pathways driving the transcription of the RUNX2 gene are triggered by the agonists Wnt—the downstream target of which is β-catenin—and bone morphogenetic proteins 2 and 4 (BMP2/4), which activate Smads 1/5/8 [15]. β-catenin—bound to the LDF1/TCF1 transcription factor—and the activated Smads form complexes on the promoter of the RUNX2 gene, stimulating its transcription [16].

With respect to BMP2/4, AMPK activation promotes the expression of BMP2/4 in osteoblasts [17,18,19]. Conversely, CK2 diminishes BMP2/4 signaling by binding to the BMP type I receptor [20]. Curiously, certain drugs designed to inhibit CK2’s kinase activity have been shown to alleviate this inhibition [20].

Estrogen activity, on the other hand, promotes RUNX2 transcription by up-regulating β-catenin signaling. This effect is downstream from the estrogen-mediated activation of endothelial nitric oxide synthase, which is observed in osteoblasts and osteocytes [21,22]. The mechanism of this is likely comparable to that observed in endothelial cells—estrogen binding induces the translocation of an N-terminally truncated ERα to the plasma membrane, where it forms a protein complex with eNOS and promotes its phosphorylation and activation via a G-protein-mediated pathway [23]. Importantly, both AMPK and Sirt1 can also boost eNOS activity; AMPK confers an activating phosphorylation on Ser-1177, whereas SIRT1 performs a deacetylation on eNOS, which enhances its activity [24,25]. The resulting production of nitric oxide (NO) can activate soluble guanylate cyclase in osteocytes and osteoblasts, and the consequent generation of cyclic GMP (cGMP) can activate both forms of protein kinase G, PKGI and PKGII [22]. The latter, for reasons not yet clear, can enhance AKT activity in osteoblasts and osteocytes [22]. AKT, in turn, via an inhibitory phosphorylation of glycogen synthase kinase-3β (GSK-3β), suppresses the ubiquitination and degradation of β-catenin, and enables it to migrate to the nucleus where it can promote RUNX2 transcription [26]. Activated AMPK can also induce the inhibitory phosphorylation of GSK-3β in these cells [27]. Furthermore, PKGII activity aids the survival of osteocytes and osteoblasts, as AKT is a well-known inhibitor of apoptosis [28]. Moreover, PKGI activation also helps ward off apoptosis through an inhibitory phosphorylation of BAD [22]. The apoptosis of osteoblasts and osteocytes is also opposed by β-catenin activity [29].

An additional mechanism whereby estrogen can support sGC activation is by the induction of cystathionine γ-lyase, an enzyme that generates hydrogen sulfide (H_2_S) [30]. H_2_S has been shown to reverse the oxidative inhibition of sGC, preserving its sensitivity to stimulation by NO [31,32] Moreover, by inducing sulfhydration of 2 cysteines in RUNX2, H_2_S can boost the transactivational activity of this transcription factor [33].

The favorable impact of intermittent treatment with the parathyroid hormone (PTH) on bone density appears to reflect the ability of cAMP/protein kinase A (PKA) signaling to inhibit GSK-3β in osteoblasts and thereby up-regulate β-catenin activity [34]. On the other hand, PKA, via the activation of the cAMP response element-binding protein (CREB), drives the expression of RANKL and suppresses that of OPG, effects that promote osteoclastogenesis [35]. The latter effect predominates when PTH signaling is strong and sustained, as during hyperparathyroidism. Intermittent mechanical loading on the bone also aids bone health via the inhibition of GSK-3β by cAMP/PKA. Loading causes fluid sheer stress on osteocytes that evokes prostaglandin E2 production; consequent autocrine activation of the EP2 receptor induces cAMP production, while also promoting Akt activity, both of which inhibit GSK-3β [5,36].

RUNX2 activity is modulated by post-translational modifications. Deacetylation of RUNX2 by Sirt1 enhances its transactivational activity [37]. AMPK confers a phosphorylation on the DNA-binding domain of RUNX2 that protects it from ubiquitination and proteasomal degradation [38]. Additionally, as we have noted, an interaction with H_2_S can also boost the transactivational activity of this transcription factor [33].

In light of the role that osteoblast autophagy plays in promoting bone mineralization and warding off apoptosis, it is notable that both AMPK and Sirt1 are well known for their up-regulatory effects on autophagy [39,40,41]. A further way in which Sirt1 exerts an anabolic effect on bones is via the inhibition of sclerostin expression at the transcriptional level [42,43]. Sclerostin is a protein produced by osteocytes that interferes with Wnt/β-catenin signaling by competitive binding to the LRP5/6 receptors that mediate Wnt signaling [44].

### 2.2. Regulation of NFATc1 Expression and Activity in Osteoclasts

The regulation of NFATc1 activity in osteoclasts is complex, involving both a priming step and a calcium-catalyzed activation and amplification step [3]. The interaction of RANK with RANKL initiates the assembly of a signaling platform, featuring TRAF6, which activates both NF-kappaB and the MAP kinases JNK and p38. This signal is amplified by a concurrent increase in reactive oxygen species (ROS) attributable to the stimulation of NOX1 activity [45]. MAP-kinase activation, in turn, activates AP-1 transcription factors, and AP-1 and NF-kappaB interact on the promoter of the NFATc1 gene to induce its transcription. NFATc1, in turn, promotes the early expression of the protein estrogen-induced gene 1 (EEIG1), which, after priming by activated RANK, forms a complex of proteins—including Bruton’s tyrosine kinase (BTK)—that promotes activating tyrosine phosphorylation of phospholipase C-γ (PLC-γ) [46,47]. Activated PLC-γ, in turn, via the formation of inositol-triphosphate, releases calcium from the endoplasmic reticulum, inducing a surge in free intracellular calcium that enables the nuclear import of NFATc1 by activating the phosphatase calcineurin, as explained below [3].

The protein complex-mediated activation of PLC-γ activation is also contingent on the activation of the tyrosine protein kinase Syk [3,48]. This requires the interaction of Syk with a plasma membrane signaling complex involving DAP12 and FcRγ; these express the immunoreceptor tyrosine-based activating motifs (ITAMs) characteristic of immunoglobulin receptors [49]. The phosphorylation of these tyrosines—likely by Fyn [50]—enables them to interact with Syk via Syk’s SH2 domain. Syk is subsequently tyrosine phosphorylated and activated by c-Src, whose activation reflects an interaction with β3 integrin [51].

NFATc1 is readily susceptible to phosphorylation by GSK-3β; this causes it to be sequestered in the cytoplasm, preventing it from influencing transcription in the nucleus [52,53]. However, calcineurin, activated by an increase in free intracellular calcium, reverses this phosphorylation, enabling NFATc1’s transport to the nucleus [54]. Moreover, via the activation of phosphatidylinositol-3-kinase and subsequently AKT, activated RANK inhibits GSK-3β activity, reinforcing the ability of NFATc1 to migrate to the nucleus [55]. Within the nucleus, NFATc1 can bind to the promoter of its own gene, accelerating its transcription; this effect is contingent of the concurrent promoter binding of an AP-1 complex containing c-Fos [51]. Hence, NFATc1 activity is boosted by an auto-amplification mechanism. The highly active NFAT1c then promotes the expression of a number of proteins required for the effective osteoclast function [13].

AMPK, Sirt1, and Nrf2 have all been shown to diminish NFATc1 activation in RANKL-treated osteoclasts. Sirt1 and Nrf2 both oppose the ROS-mediated amplification of NF-kappaB and MAP kinase activation via antioxidant effects. Nrf2 does so via phase 2 induction of a range of antioxidant enzymes, and of the rate-limiting enzyme for glutathione synthesis [56,57]. Additionally, via the induction of heme oxygenase-1 (HO-1), Nrf2 induces the direct inhibition of NOX1; the carbon monoxide evolved by heme oxygenase activity has been shown to inhibit NOX1 [58]. Sirt1 promotes the induction of another set of antioxidant enzymes—including HO-1and catalase—that are transcribed in response to the FOXO1 transcription factor; Sirt1 enables this by removing an acetylation from FOXO1 that blocks its efficacy in this regard [59]. Importantly, Sirt1 also diminishes NF-kappaB transcriptional activity by deacetylating its p65 component [60].

AMPK has been shown to inhibit RANKL-mediated osteoclastogenesis [61,62,63]. Its mechanism in this regard has not been established, but one credible possibility is that it suppresses Syk activation by promoting its interaction with the tyrosine phosphatase SHP-1; this phenomenon has been reported in mast cells [64]. SHP-1 activity is known to oppose osteoclastogenesis by opposing Syk activity in osteoclasts [65].

In osteoclasts, CK2 has been shown to amplify RANK-mediated AKT activation, likely because CK2 can confer an inhibitory phosphorylation on PTEN, an antagonist of AKT activation [66,67]. This effect enhances RANK’s ability to inhibit GSK-3β, preventing the inhibitory phosphorylation of NFATc1 [66].

Inflammation-induced bone loss, such as that associated with rheumatoid arthritis or periodontis, involves cytokine-mediated osteoclast activation; tumor necrosis factor-α (TNFα) may be the primary mediator in this regard [68,69]. This effect is RANKL independent, though RANKL signaling can provide potentiation. RANKL and TNFα activate a common target, NF-kappaB, to promote the expression of NFATc1; hence, it is not surprising that the activation of Sirt1 or of AMPK (upstream from Sirt1) have the potential to suppress TNFα-mediated osteoclastogenesis [61,70,71,72]. Systemic inflammatory disorders often compromise bone health indirectly by necessitating the administration of clinical glucocorticoids. The initial response to supraphysiological glucocorticoid activity in bones is an up-regulation of osteoclastic activity—in part, reflecting a suppression of osteoblasts OPG production [73,74]. During longer term therapy, a suppression of osteoblast differentiation and an up-regulation of apoptosis in osteoblasts and osteocytes also contributes to the loss of bone mass associated with glucocorticoid treatment [74,75,76,77].

### 2.3. Modulation of RANKL and OPG Secretion by Osteoblasts and Osteocytes

The extent to which osteoblasts and osteocytes produce RANKL and its functional antagonist osteoprotegerin (OPG)—which serves as a decoy receptor for RANKL [78]—is also a key determinant of osteoclastogenesis.

While PKA activation in osteoblasts/osteocytes can exert an anabolic effect on bones by boosting the beta-catenin signal, PKA activity can also promote bone catabolism. PKA, via the activation of the cAMP response element-binding protein (CREB), drives the expression of RANKL and suppresses that of OPG, effects that promote osteoclastogenesis [35]. The latter effect predominates when PTH signaling is strong and sustained, as during hyperparathyroidism.

AMPKα2 acts on osteoblasts to diminish their production of RANKL, while boosting their production of the RANKL antagonist OPG; this effect might reflect an opposition to CREB signaling [79,80,81]. The ability of PTH to drive the expression of RANKL has been attributed to cAMP/PKA/CREB signaling that requires CTRC2 as a coactivator for CREB; notably, AMPK has been reported to antagonize CTRC2 activity by conferring a phosphorylation on it that causes its exclusion from the nucleus [82,83,84]. Whether CTRC2 participates in the PTH-mediated suppression of OPG should be investigated. Conceivably, AMPK agonists, such as berberine, could make the impact of PTH and mechanical loading on bones more uniformly positive. β-catenin activity, independent of its impact on RUNX2, also increases OPG production by osteoblasts and osteocytes, as the β-catenin/TCF complex binds the promoter of the OPG gene and drives its transcription [85,86].

## 3. Nutraceutical Measures for Bone Health

The preceding discussion enables us to predict that nutraceuticals that activate AMPK, Sirt1, Nrf2, and sGC, or that inhibit CK2, could favorably influence bone density by promoting RUNX2 activity and autophagy—while also suppressing apoptosis—in osteoblasts and osteocytes. Such agents could also be expected to oppose NFATc1 activity, thereby decreasing osteoclastogenesis and osteolysis.

With respect to AMPK, the prototypical pharmaceutical activator of AMPK, metformin, has been associated with a lower risk for fracture and higher bone density in diabetics using this drug, as opposed to not using it [87,88,89,90,91]. Analogously, metformin is protective in rodent models of bone loss [72,92,93,94,95]. The nutraceutical berberine, long used in China for treatment of type 2 diabetes and dyslipidemias, is known to activate AMPK in a manner similar to metformin [96,97,98,99,100,101]. Berberine has been reported to exert bone protective effects in a number of rodent models of bone loss [102,103,104,105,106,107].

With respect to Sirt1, there is a growing list of nutraceuticals—aside from resveratrol, whose pharmacokinetics in humans render it of dubious clinical utility [108,109]—which have been reported to increase Sirt1 expression or activity in various contexts. Berberine and other AMPK activators do so, owing to the induction of nicotinamide phosphoribosyltransferase (NAMPT), which is rate-limiting for the re-synthesis of Sirt1’s obligate substrate NAD+ [110,111,112,113,114,115]. Moreover, NAMPT induction not only boosts Sirt1 activity by increasing NAD+, but also by decreasing cellular levels of nicotinamide, a product of Sirt1 activity that acts as an end-protein inhibitor of this enzyme [116]. The nutraceutical nicotinamide riboside (NR) offers an alternative strategy for increasing cellular NAD+, as it can function as a substrate for biosynthesis of this compound [117]. Melatonin promotes Sirt1 expression at the transcriptional level, likely via the activation of the Bmal1 transcription factor [118,119,120,121]. Ferulic acid—likely a key mediator of the health benefits of dietary anthocyanins and whole grains—also increases Sirt1 expression at the mRNA level [122,123,124,125,126,127]. N1-methylnicotinamide (MNA), a natural metabolite of nicotinamide with anti-inflammatory activity, prolongs Sirt1 half-life, possibly by opposing a JNK-mediated phosphorylation of Sirt1 that promotes its proteasomal degradation [128,129,130]. Supplemental glucosamine may have the potential for activating Sirt1, as the O-GlcNAcylation of Sirt1 has been reported to boost Sirt’s deacetylase activity [131,132]. Additionally, the key active component of black cumin seed oil, thymoquinone, boosts Sirt1 activity, likely by promoting the conversion of NADH to NAD+ when reduced by NAD(P)H quinone oxidoreductase (NQO1) [133,134,135,136,137,138]. The favorable effects of melatonin, ferulic acid, NR, and glucosamine on bone density have been reported in rodent studies, while the effects of thymoquinone and MNA on bones have received minimal, if any, attention [125,139,140,141,142,143,144,145,146,147,148,149,150,151,152,153,154,155,156,157,158,159].

A number of phytochemicals, as well as the physiologically essential cofactor lipoic acid, have shown utility for boosting Nrf2 activity. Lipoic acid, thymoquinone, and the sulforaphane generated from cruciferous vegetables do so by the covalent interaction with Keap1, the protein that retains Nrf2 in the cytoplasm and promotes its proteasomal degradation [160,161,162,163,164]. Melatonin boosts the expression of Nrf2 via the Bmal1 transcription factor [165,166]. The xanthophyll carotenoid astaxanthin can also enhance the expression of Nrf2, possibly via the interaction with the aryl hydrocarbon receptor [167,168,169,170,171,172,173,174]. Lipoic acid, astaxanthin, and—as noted—melatonin have shown utility in rodent models of bone loss [175,176,177,178,179,180,181,182,183].

Cinaciguat, a direct activator of the oxidized form of sGC, has been shown to protect bone density in ovariectomized mice [21]. In concentrations that are two orders of magnitude higher than the physiological level, the B vitamin biotin can directly activate the native form of sGC, promoting cGMP production [184]. In rodent studies, ample oral biotin intakes have been shown to boost cGMP levels [185,186,187]. Since high-dose biotin is clinically well tolerated, it might be considered as a strategy for aiding bone density [188]. It does not appear to have been studied in that regard in rodents, however. The one known clinical drawback of high-dose biotin is that it can interfere with certain lab assays that employ biotinylated reagents; hence, the discontinuation of biotin for at least several days may be prudent when lab tests are planned [189].

With respect to the inhibition of CK2, a range of flavonones—including quercetin, myricetin, fisetin, kaempferol, luteolin, and apigenin—have been shown to inhibit CK2’s kinase activity in high nanomolar concentrations that might be clinically relevant when high-absorption forms of these flavonols are ingested [190,191,192,193]. A number of studies have found that quercetin favorably influences bone density in rodent models of bone loss [194,195,196,197,198,199]. Derivatized or nanoparticulate preparations of quercetin designed for optimal absorption are available as nutraceuticals [200,201,202,203]. Whether quercetin can prevent the inhibitory interaction between CK2 and the BMP type-I receptor remains to be determined.

In addition to the nutraceuticals previously mentioned, taurine has shown positive effects on bone density in rodents [204,205,206,207,208,209]. In vitro, taurine has been reported to suppress sclerostin production by osteocytes—which can curiously synthesize their own taurine—and to decrease ROS levels in activated osteoclasts [210,211]; whether these effects are clinically relevant is unclear, as high concentrations of taurine were employed in these cell culture studies. Taurine promotes the induction of cystathionine γ-lyase (CSE) in vascular endothelial cells, and the possibility that it, similar to estrogen, does so in osteoblasts can be entertained [212,213,214]. As noted, the H_2_S that CSE produces has a favorable impact on osteoblastic activity. N-acetylcysteine (NAC), which generates the cysteine that serves as CSE’s substrate, could presumably enhance H_2_S production in osteoblasts, and has shown favorable effects on rodent models of bone loss [215,216,217,218]. NAC might also aid the maintenance of bone density by boosting osteoclast glutathione synthesis, and thereby opposing the up-regulatory effect of ROS on RANKL signaling. Indeed, in a very small pilot trial, the supplementation of recently post-menopausal women with 2 g of NAC daily, as an adjuvant to calcium/vitamin D supplementation, was associated with a trend toward a greater reduction in the marker of bone resorption serum C-telopeptide than in the placebo group; sadly, this lead has not been followed up [219].

In addition to the nutraceuticals discussed above, there are many other phytochemicals with the potential for boosting Sirt1, AMPK, or Nrf2 activities. As examples, urolithin A, a bacterial metabolite of pomegranate ellagitannins thought to mediate the protective properties of pomegranate juice, has recently been reported to increase Sirt1 expression and NAD+ levels [220,221,222,223]. Compounds in bitter melon (Momordica charantia), a food traditionally used in diabetes management in southeast Asia, have been found to boost AMPK activity by activating its upstream kinase Ca^+2^/calmodulin-dependent kinase kinase-β [224]. Additionally, a wide range of phytochemicals have some potential as Nrf2 activators [225]. The agents highlighted in this essay are distinguished by the fact that they are readily available in nutraceutical form and have, to some degree, been clinically employed.

## 4. Nutraceutical Control of Systemic Inflammation

With respect to the loss of bone mass associated with systemic inflammation, it stands to reason that nutraceuticals measures, which can quell inflammation, may be of clinical benefit—not only by decreasing the production of pro-inflammatory cytokines that boost osteoclasts activity, but also by decreasing the clinical need for glucocorticoid (GC) therapy. The joint activation of the transcription factors NF-kappaB and AP-1 plays a key role in promoting the macrophage and monocyte expression of TNF-α and other pro-inflammatory hormones at the transcriptional level [226]. Sub-optimal Sirt1 activity often collaborates with oxidative stress in boosting NF-kappaB and AP-1 activity; the oxidant-driven activation of JNK and p38 MAP kinases mediates AP-1 activation [226,227,228]. Hence, nutraceutical measures that boost Sirt1 activity and control oxidant stress—such as Nrf2 activators and NAC, a glutathione precursor– have the potential for the control of inflammation-driven bone loss. NOX2-dependent NADPH oxidase activity importantly contributes to oxidant production in macrophages, and the phycochemical phycocyanobilin, a chemical relative of bilirubin that functions as a light-harvesting chromophore in cynobacteria and some blue-green algae, has been found to inhibit this activity by mimicking the physiological antioxidant role of intracellular free bilirubin [229,230]. This may explain why spirulina, an exceptionally rich source of phycocyanobilin, was found to be highly protective in a P. gingivalis-driven rat model of periodontal inflammation and alveolar bone loss [229,231]. Analogously, the oral administration of spirulina or its chief protein phycocyanin (covalently linked to phycocyanobilin) have shown marked efficacy in rodent models of inflammatory arthritis and other inflammatory conditions [232,233,234,235].

Curiously, there is evidence that nutraceuticals capable of activating Sirt1 can oppose the ability of GCs to impede osteoblast maturation and induce bone loss in rodents; this has been demonstrated for ferulic acid, melatonin, berberine, and nicotinamide mononucleotide [125,155,236,237]. While these effects might be expected, owing to Sirt1’s ability to work in various ways to promote RUNX2 activity, it is conceivable that it works more proximally to interfere with GC signaling in osteoblasts and their precursors. In this regard, there is recent evidence that the negative impact of high-dose GCs on osteoblast maturation and function may be mediated, in large part, by the transcriptional induction of PPARγ, which, in turn, induces the expression of secreted frizzled-related protein 5 (SFRP5), an antagonist of the Wnt signaling crucial to osteoblast induction [238]. Moreover, Sirt1 activity has been shown to oppose PPARγ expression and activity in a pre-osteoblast cell line; it has previously been established that Sirt1 opposes PPARγ-driven transcription in adipocytes [239,240]. (These considerations are evidently pertinent to the adverse impact of thiazolidinedione therapy on bone, as these agents serve as PPARγ agonists [241].) Importantly, the anti-inflammatory effects of GCs do not appear to be mediated by PPARγ [242]. Importantly, the anti-inflammatory effects of GCs do not appear to be mediated by PPARg [Evidently, nutraceuticals that can exert anti-inflammatory effects, while reducing the toxicity of GCs to the bone, might prove to be valuable complements to the therapy of autoimmune disorders.

## 5. Optimal Intakes of Certain Essential Vitamins and Minerals also Aid the Maintenance of Bone Density

Insuring adequate or ample intakes of a number of vitamins and minerals could be expected to complement the benefits of the more novel strategies suggested in this essay. Vitamin D aids bone health not only by helping to prevent secondary hyperparathyroidism, but also because calcitriol, produced from circulating 25-hydroxyvitamin D by 25-hydroxyvitamin D 1-α-hydroxylase (CYP27B1) in osteoblasts, complements the transcriptional activity of RUNX2 when bound to the vitamin D receptor; most notably, it collaborates with RUNX2 in promoting the expression of osteocalcin (OC), a crucial mediator of hydroxyapatite deposition [243,244,245,246,247]. Hence, bioavailable plasma levels of 25-hydroxyvitamin D tend to positively correlate with bone density [248,249,250,251]. Vitamin K2 (the bacterially produced form found in fermented milk and soy milk products, which achieves greater systemic distribution than the more hepatotropic vitamin K1) is thought to promote bone health by inducing γ-carboxylations of the bone matrix proteins OC and periostin, thereby improving their function in the bone [252,253]. Curiously, the beneficial impact of supplemental vitamin K2 on fracture risks in post-menopausal women appears to be disproportionate to its modest effect on bone density—possibly reflecting a favorable impact of vitamin K2 on bone flexibility [254,255]. Ample dietary intakes or increased serum levels of magnesium and zinc have been associated with greater bone density or a lower risk of fractures [256,257,258]. (Perhaps, surprisingly, higher intakes of calcium, while associated with a modestly greater bone density post-menopausally, do not appear to influence fracture risks [259,260]). Rodent studies suggest that increased intakes of manganese or silicon may have a positive impact on bone density [261,262]. Diets habitually high in natural potassium also positively correlate with bone density, likely because the organic counteranions ingested with the potassium are metabolized to bicarbonate, and hence exert an alkalinizing effect that opposes osteolysis [263,264,265]. Conversely, dietary sulfhydryl amino acids are metabolized to generate sulfate, and hence are acidifying; this suggests that supplemental NAC might have its most favorable net impact on bone health in the context of a diet naturally high in potassium [266]. Comprehensive vitamin–mineral supplementation, particularly in the context of sub-optimally nutritious diets, could be expected to favorably impact bone density and fracture risks [267].

## Data Availability

Not applicable.
